# 1667. Patterns of Inpatient Antibiotic Use by Race and Ethnicity at US Children's Hospitals

**DOI:** 10.1093/ofid/ofad500.1500

**Published:** 2023-11-27

**Authors:** Bethany Wattles, Jeffrey I Campbell, Yana Feygin, Kahir S Jawad, Michelle D Stevenson, Deborah Davis, Jennifer Porter, V Faye Jones, Michael J Smith

**Affiliations:** University of Louisville School of Medicine, Louisville, Kentucky; Boston Medical Center, Boston, Massachusetts; University of Louisville, Louisville, Kentucky; University of Louisville, Louisville, Kentucky; Norton Children's Medical Group and University of Louisville, Louisville, Kentucky; Norton Children's and University of Louisville, Louisville, Kentucky; Norton Children's and University of Louisville, Louisville, Kentucky; Norton Children's and University of Louisville, Louisville, Kentucky; Duke University, Durham, North Carolina

## Abstract

**Background:**

Racial and ethnic variations in antibiotic prescribing to children are well-reported in outpatient settings. However, little is known about racial prescribing patterns in hospital settings. We sought to describe national inpatient antibiotic utilization among children by race and ethnicity.

**Methods:**

We utilized Pediatric Health Information System (PHIS) data from 49 children’s hospitals across the US. All inpatient and observation-status visits from 2022 for children < 18 years were included. An “antibiotic visit” was defined as any inpatient admission in which an oral, parenteral, or intramuscular antibiotic was prescribed. Antibiotic visits, severity, length of stay, and cost of stay were stratified by patient race and ethnicity (as collected by each hospital). Diagnoses and severity were classified by All Patients Refined Diagnosis Related Groups (APR-DRG).

**Results:**

Antibiotic visits by race-ethnicity ranged from 38.4% (Non-Hispanic [NH] Black children) to 45.0% (NH American Indian) (**Table 1**). NH White children made up the largest group (45.2% of all visits) and had an antibiotic visit rate of 42.4%, followed by Hispanic (27.1% of all visits) and NH Black (19.2%). Antibiotic visits for children < 2 months ranged from 19% (Asian) to 39.4% NH American Indian. The largest gap in antibiotic visits between NH White and NH Black children was in ages 5 to 11 years (49% and 40.6%, respectively). Differences between NH White and NH Black children were highest in the northeast (44.7% vs. 36.6%, respectively), and lowest in the south (40.1% vs. 38.9%, respectively). In NH White and Hispanic children, antibiotic visits decreased with increasing income and Child Opportunity Index; for NH Black children, a reverse relationship was observed. Severity was similar for NH White, Hispanic and NH Black children. NH Black children had the highest proportion of stays >14 days (6.6%) but lower cost of stays. Antibiotic visits and diagnoses of interest are reported in Table 2.

**Table 1.**
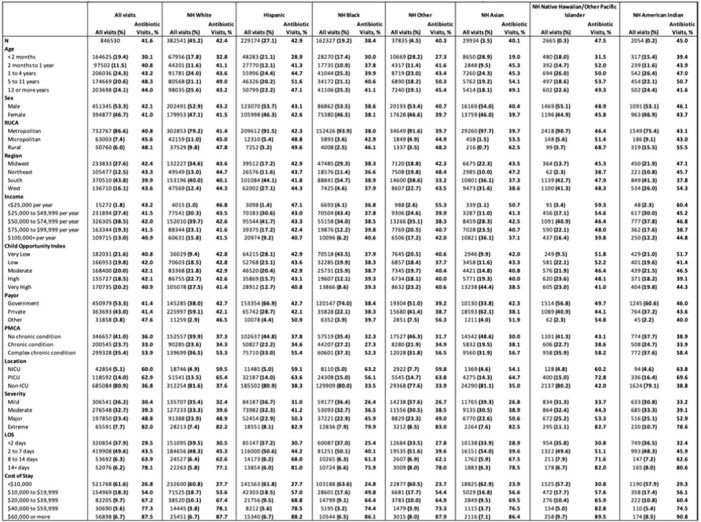
Demographics and Antibiotic Visits by Race and Ethnicity. NH = Non-Hispanic; Child Opportunity Index (a neighborhood measure of resources and conditions for healthy development) and Income were assigned using patient zip code.

**Table 2.**
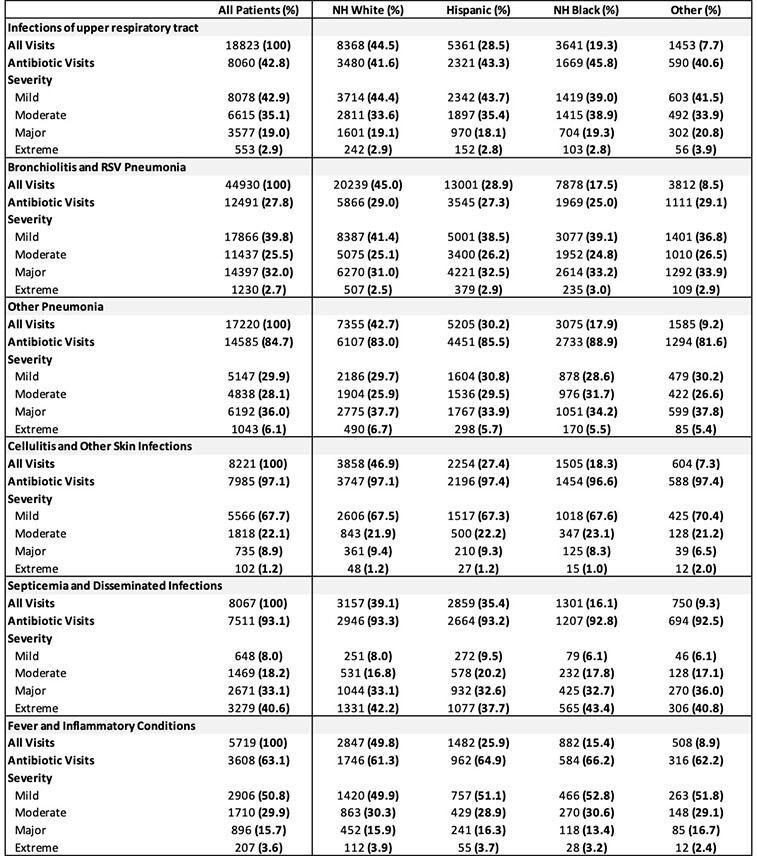
Antibiotic Visits and Outcomes by All Patients Refined Diagnosis Related Groups (APR-DRG). All Visits, Antibiotic Visits = row %; Severity = column %

**Conclusion:**

Antibiotic utilization in children’s hospitals differs by race and ethnicity. Antibiotic stewardship programs should stratify antibiotic use data by race-ethnicity and examine policies and practices that may contribute to disparities in treatment for hospitalized children.

**Disclosures:**

**Bethany Wattles, PharmD, MHA**, Merck: Grant/Research Support **Michael J. Smith, M.D., M.S.C.E**, Merck: Grant/Research Support|Pfizer: Grant/Research Support

